# Selective Wettability–Driven Evaporation-Enhanced Redox Cycling for Robust and Ultrasensitive Detection of Viral Particles

**DOI:** 10.1002/smtd.202502229

**Published:** 2026-01-18

**Authors:** Pouya Soltan Khamsi, Shubhada K Chothe, Suresh V Kuchipudi, Aida Ebrahimi

**Affiliations:** 1 Department of Electrical Engineering, The Pennsylvania State University, University Park, Pennsylvania, USA; 2 Materials Research Institute, The Pennsylvania State University, University Park, Pennsylvania, USA; 3 Department of Infectious Diseases and Microbiology, School of Public Health, University of Pittsburgh, Pittsburgh, Pennsylvania, USA; 4 Center for Vaccine Research, University of Pittsburgh, Pittsburgh, Pennsylvania, USA; 5 Department of Biomedical Engineering, The Pennsylvania State University, University Park, Pennsylvania, USA

**Keywords:** electrochemical biosensor, redox cycling, selective wettability, superhydrophobic surface, virus detection

## Abstract

Timely detection of viral infections, particularly in settings outside centralized laboratories, is essential for controlling outbreaks. Small electrochemical biosensors are appealing because they are fast, require microliter sample volumes, and can be integrated into portable devices. However, shrinking the sensor area often weakens the signal, making it difficult to detect low viral loads. We previously showed that combining redox cycling with controlled droplet evaporation can boost the signal. Yet, this method faces signal variability due to user-dependent droplet placement. Here, we introduce a selective-wettability, evaporation-enhanced redox cycling (SW-E2RC) device that passively centers and pins the droplet on the sensing area, improving both signal strength and reproducibility. The chip combines a wettable sensing zone surrounded by water-repellent micropillars that guide the droplet into place and stabilize it during evaporation, concentrating redox-active species over the electrodes. Using SARS-CoV-2 and avian influenza H5N1 as model pathogens, we show that SW-E2RC reduces the LOD to 9.2 × 10^3^ copies/mL, corresponding to a ~10^3^-fold improvement in sensitivity compared with E2RC. This platform can be adapted to various capture probes and targets, enabling more sensitive and reliable point-of-need viral diagnostics.

## 1 ∣ Introduction

Infectious diseases remain a persistent challenge in global health, evidenced by the COVID-19 pandemic and the ongoing appearance of highly transmissible variants such as Omicron, along with zoonotic threats like highly pathogenic avian influenza (HPAI) H5N1 virus [1-5]. Early detection, especially at low viral loads during pre-symptomatic stages and asymptomatic infections, is crucial for preventing widespread outbreaks [6-8]. However, current gold-standard molecular diagnostics, such as reverse transcription polymerase chain reaction (RT-PCR) [9] assays, while highly sensitive, require specialized laboratories, trained personnel, and are time-consuming processes [10]. This limits their effectiveness for rapid, on-site detection, particularly in resource-limited or field settings [11].

Electrochemical biosensors offer a promising alternative for point-of-care diagnostics by rapidly and selectively detecting target analytes through converting biorecognition events into measurable electrical signals [12-15]. These devices are ideal for portable and field testing becasue of their low-cost, simple operation, and ability to fit into compact systems [16-18]. Their small sample volume requirements [16, 19], fast response times [20, 21], and compatibility with miniaturization [22, 23] make them perfect for detecting pathogens directly at the patient’s location, reducing diagnostic turnaround time and enabling quicker intervention [24, 25]. However, performance can decline as devices are made smaller. Reducing the sensing area often worsens diffusion limitations, where the transport of analyte molecules to the electrode surface becomes the limiting factor [26]. This can lead to weaker electrochemical signals and lower sensitivity, especially when detecting trace levels of analytes like viral particles during early infections or in asymptomatic carriers [27, 28]. To address these issues, signal amplification strategies such as catalytic redox cycling [29], nanostructured electrodes [30], and microfluidic confinement [31] have been developed to overcome mass-transport limitations and restore sensitivity in miniaturized biosensors.

Our group recently showed that evaporation-enhanced redox cycling (E2RC) can detect viral particles at attomolar concentrations in about 20 minutes by surpassing the diffusion limit typical of miniaturized electrochemical sensors through continuous signal readout in evaporating droplets [32]. The process of droplet evaporation further concentrates analytes over time, increasing the local concentration at the sensing interface and enhancing mass transport beyond passive diffusion [33, 34]. Continuous, real-time monitoring of signal during evaporation captures the increase of both the redox probe concentration and the electrode surface blockage caused by accumulating viral particles. However, the E2RC platform depends on precise manual droplet placement to align over the sensing area. Improper droplet alignment can reduce reproducibility and sensitivity, especially in field conditions where precise pipetting may be challenging.

Studies have shown that engineering surface wettability can address these issues. Zhang et al. demonstrated that ferrofluid-infused surfaces, combined with magnetic actuation, can precisely guide and localize droplets using a wetting ridge-assisted programmable mechanism to control droplet position, thereby improving reproducibility in point-of-care diagnostic platforms [35]. In another study, Wallace et al. developed superhydrophobic (SHP) surface-enhanced Raman spectroscopy (SERS) substrates using deterministic (stochastic) pillar arrays functionalized with silver colloids. Their platform enabled over 100-fold analyte concentration by evaporating droplets on the textured surface, achieving a limit of detection of 2.9 *pM* mitoxantrone dihydrochloride, showing engineered micro-and nano-scale topography effectively promoted analyte localization and provided reproducible SERS signals across multiple samples [36]. A similar strategy was reported by De Angelis et al., by engineering photonic–plasmonic nanostructures that leveraged evaporation-driven flows to concentrate analytes into nanoscale “hot spots” for label-free single-molecule detection. Their approach highlights how surface design and droplet dynamics can work together to boost local analyte concentration and detection sensitivity [33]. On the other hand, recent studies suggest that introducing a mildly slippery [37], low-hysteresis coating [38] within the sensing zone could further regulate receding evaporation and promote centripetal solute enrichment. Such coating suppresses contact-line pinning and enables smoother, axisymmetric recession, which may enhance evaporation-driven analyte concentration without affecting the droplet’s self-alignment on the surrounding superhydrophobic region [39]. These findings highlight the potential of engineered/selective wettability [40-42] to improve the sensitivity and reliability of biosensing systems, including electroanalytical devices. The desired limit of detection for both SARS-CoV-2 and H5N1 is around 10^2^–10^3^ copies/mL, comparable to RT-qPCR sensitivity and significantly better than typical antigen tests [43-47]. Across electrochemical and field effect transistor (FET)-based sensors for SARS-CoV-2, reported limits of detection (LODs) range from ~10^5^ to 10^5^ copies/mL. Graphene-FETs have demonstrated ~65 to 2.42 × 10^2^ copies/mL on clinical swabs [48, 49]. while electrochemical assays vary from about 2.0 × 10^2^ copies/mL (amplification-free supersandwich RNA) to roughly 6.9 × 10^3^ copies/mL (amplification-free antisense ePAD), and as low as 10^3^ copies/mL when RCA amplification is applied [50-52]. Notably, amplification-free nucleic-acid electrochemical impedance spectroscopy assays have reported LODs on the order of ~10^5^ copies/mL, underscoring the challenge of achieving high sensitivity without preamplification in miniaturized electrochemical platforms [53].

In this work, we address the signal variability of E2RC – caused by manual droplet placement – by engineering the device surface to exhibit selective wettability, with superhydrophobic regions surrounding a deliberately hydrophilic sensing zone. A hydrophilic “landing pad” embedded within the superhydrophobic background guides droplets to spontaneously confine to the sensing area through capillary forces and contact-line pinning, ensuring auto-localization during evaporation. By coupling selective wettability with E2RC, the SW-E2RC platform achieves reproducible droplet alignment, passive particle concentration, and enhanced mass transport to the electrodes. This integration improves sensitivity, reduces the detection limit by more than three orders of magnitude, and removes the need for manual positioning. Testing with SARS-CoV-2 (< 200 nm) and larger H5N1 (~1 μm) viruses showed a size-dependent response linked to the sub-micrometer sensing gap, with the highest sensitivity observed for particles closely matching the device’s geometry. Measurements of dynamic light scattering and zeta potential indicated similar negative surface charges for both viruses, suggesting that differences in sensitivity were mainly driven by size-related steric accessibility. These findings highlight SW-E2RC as a robust, adaptable, and field-ready diagnostic platform capable of detecting pathogens sensitively in microliter-scale samples, offering a framework for customizing device geometry to a wide range of biological targets in future biosensor designs.

## 2 ∣ Results and Discussion

### 2.1 ∣ Droplet-Repelling Zone: Superhydrophobic Surface Design

**FIGURE 1:**
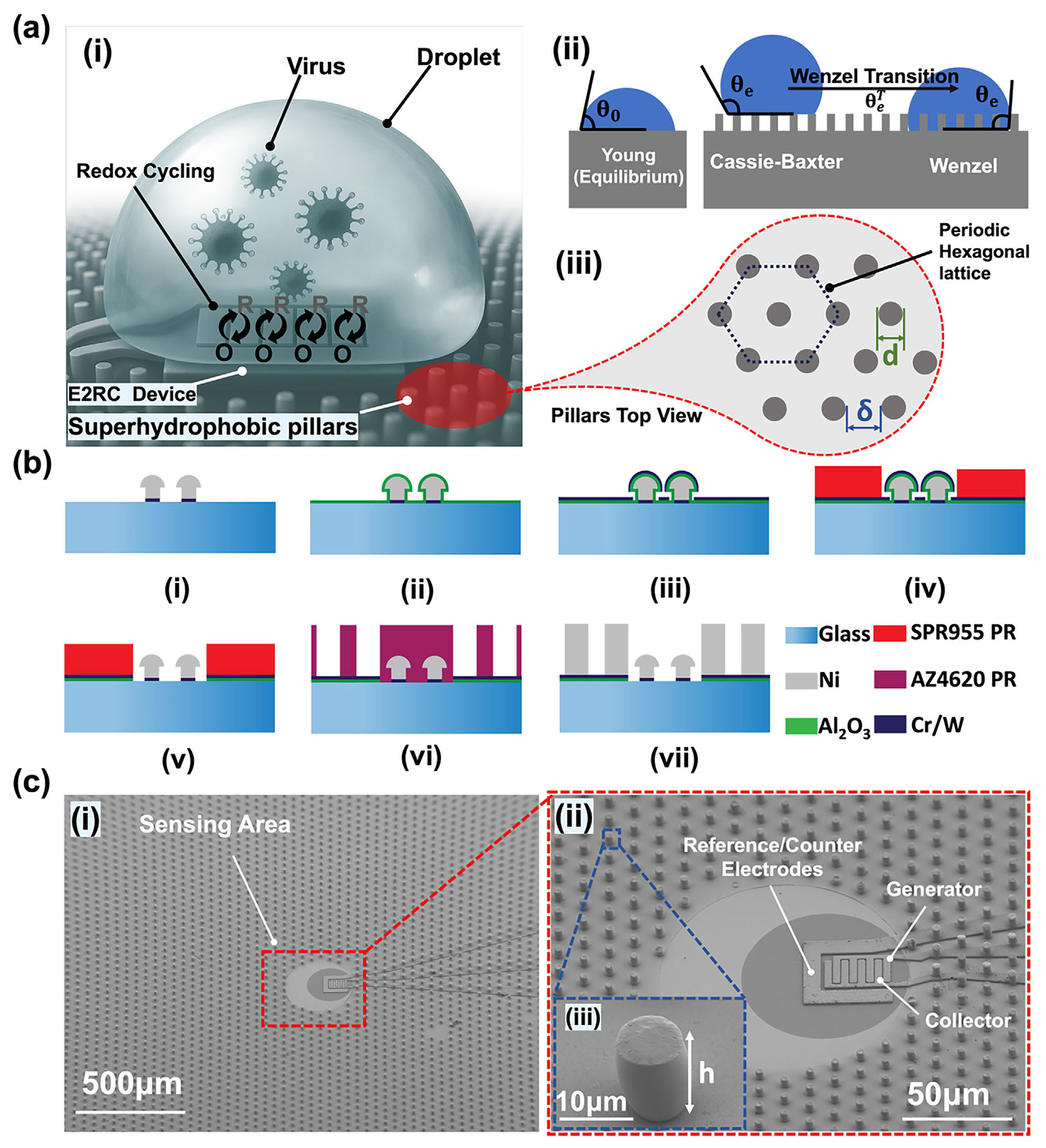
Superhydrophobic (SHP) Surface Design and Integration with the Redox Cycling Platform. (a) Conceptual framework for SW-E2RC devices for ultrasensitive detection of viral particle. (i) Illustration of a spherical droplet on a micropillar-patterned SHP surface showing minimal solid–liquid contact and dominant surface tension, enabling stable confinement during evaporation. Image generated using ChatGPT (OpenAI, 2025). (ii) Schematic showing equilibrium contact angle $\theta_{0}$ defined by Young’s equation. (iii) Top view of engineered micropillar array on a hexagonal lattice, where d is pillar diameter and δ is interpillar spacing, used to define solid fraction ($\phi_{s}$) and optimize the surface wetting properties. (b) Fabrication workflow for the integrated SW-E2RC device. (i) Baseline E2RC with interdigitated Ni microelectrodes fabricated on a Cr/W seed layer. (ii) Deposition of a conformal 100 nm Al_2_O_3_ dielectric layer by ALD for electrical isolation. (iii) Cr (10 nm)/W (100 nm) bilayer deposited as a hard mask and electroplating seed. (iv) Photolithography is used to open the sensing region and contact pads. (v) ICP-RIE used to etch exposed Cr/W and Al_2_O_3_, preserving isolation elsewhere. (vi) Patterning of AZ4620 photoresist molds for micropillar fabrication. (vii) Nickel electroplating into molds to form SHP pillars with 7μm diameter and ~12μm height. (c) SEM images of a complete SW-E2RC device. (i) An image showing SHP pillars surrounding the central sensing zone. (ii) A zoomed-in view of the generator and collector electrodes within the sensing region. (iii) The inset shows a high magnification image of a pillar with a uniform height, consistent with the design parameters.

In the first generation of E2RC devices, the users had to manually align each droplet to nearly the exact sensor location, which introduced variability and limited the platform’s reliability for real-world diagnostics [32]. To fix this, the proposed SW-E2RC device combines the sensing unit with an engineered micropillar-based SHP surface (Figure 1a (i)). Most of the device is covered with a micropillar-based SHP region, but the central sensing zone is intentionally left unstructured and hydrophilic to allow robust sensing through redox cycling. This difference in surface wettability is a key design feature: the SHP region repels the droplet and guides it toward the hydrophilic zone, where it becomes pinned and stays centered during evaporation and sensing.

Superhydrophobicity is a surface phenomenon where a liquid droplet adopts a nearly spherical shape when it contacts a textured surface. It is characterized by a high apparent contact angle (≥ 150°) and low contact angle hysteresis [33, 54-56]. This happens because of reduced adhesion between the liquid and solid, which allows the droplet to stay suspended over a combination of solid and air [57]. Such surfaces exhibit excellent water repellency and are especially useful in applications that requiring droplet confinement, self-cleaning, or improved transport properties [58, 59]. In biosensing, SHP surfaces enable controlled evaporation of microdroplets while minimizing undesirable wetting or pinning effects [60]. When a highly diluted analyte solution is placed on a carefully engineered SHP surface, the droplet maintains a quasi-spherical shape during evaporation, undergoing a self-similar geometric transformation [19, 33, 61]. This process ensures that the analyte becomes increasingly concentrated without spreading over a large area [62]. Ultimately, the droplet collapses into a confined region precisely aligned with the sensor’s active area, improving detection sensitivity even at attomolar concentrations [63].

We evaluated the superhydrophobic behavior of the micropillar array through contact angle analysis and droplet morphology tracking. The surface’s wettability is governed by the equilibrium contact angle represented by $\theta_{0}$, characterizes the interaction between a liquid droplet and a solid surface under static conditions, as shown in Figure 1a (ii). Young’s equation describes this angle [64]:

(1) $$cos\theta_{0}=\frac{\gamma_{SV}-\gamma_{SL}}{\gamma_{LV}}$$

where $\gamma_{SV}$, $\gamma_{SL}$, and $\gamma_{LV}$ are the interfacial tensions between the solid–vapor, solid–liquid, and liquid–vapor phases, respectively. We operated in the capillarity-dominated regime, characterized by a dimensionless Bond number Bo, which compares gravitational forces to surface tension forces:

(2) $$Bo=\frac{\rho \times g\times R^{2}}{\gamma_{LV}}\ll 1$$

where ρ is the density of the liquid, g is the gravitational acceleration, and R is the radius of the spherical droplet before deposition. Under these conditions, surface tension dominates over gravity, and the droplet to assume a quasi-spherical shape driven by interfacial energetics. Hence, Young’s relation remains applicable in our case. In our system, assuming a water droplet of 1.5 μL volume, the equivalent spherical radius is approximately R≈1.1.mm. By considering $\rho =1000\;kg∕m^{3}$ and $g=9.81\;m∕s^{2}$, and $\gamma_{LV}=0.072\;N∕m$, we calculated Bo≈0.14 [65, 66]. This indicates that our experiments are within the capillary regime, where surface tension dominates over gravity.

However, while Young’s equation strictly applies to smooth and chemically uniform surfaces, engineered rough surfaces (such as micropillar arrays) often behave differently. This differnece occurs because of air pockets beneath the droplet or complete liquid penetration into the surface texture, resulting in complex wetting states like Cassie-Baxter and Wenzel regimes [67]. We designed and fabricated a series of cylindrical pillars on a periodic hexagonal lattice, as shown in Figure 1a (iii), with different pitch, diameter, and height. Based on the Cassie–Baxter model [68], the apparent contact angle ($\theta_{e}$) on a structured surface is defined by:

(3) $$os\theta_{e}=-1+\phi_{s}\;(cos\theta_{0}+1)$$

where $\phi_{s}$ is the solid fraction for this layout, calculated as:

(4) $$\phi_{s}=\frac{\pi d^{2}}{2\sqrt{3}{\left(d+\delta \right)}^{2}}$$

**FIGURE 2:**
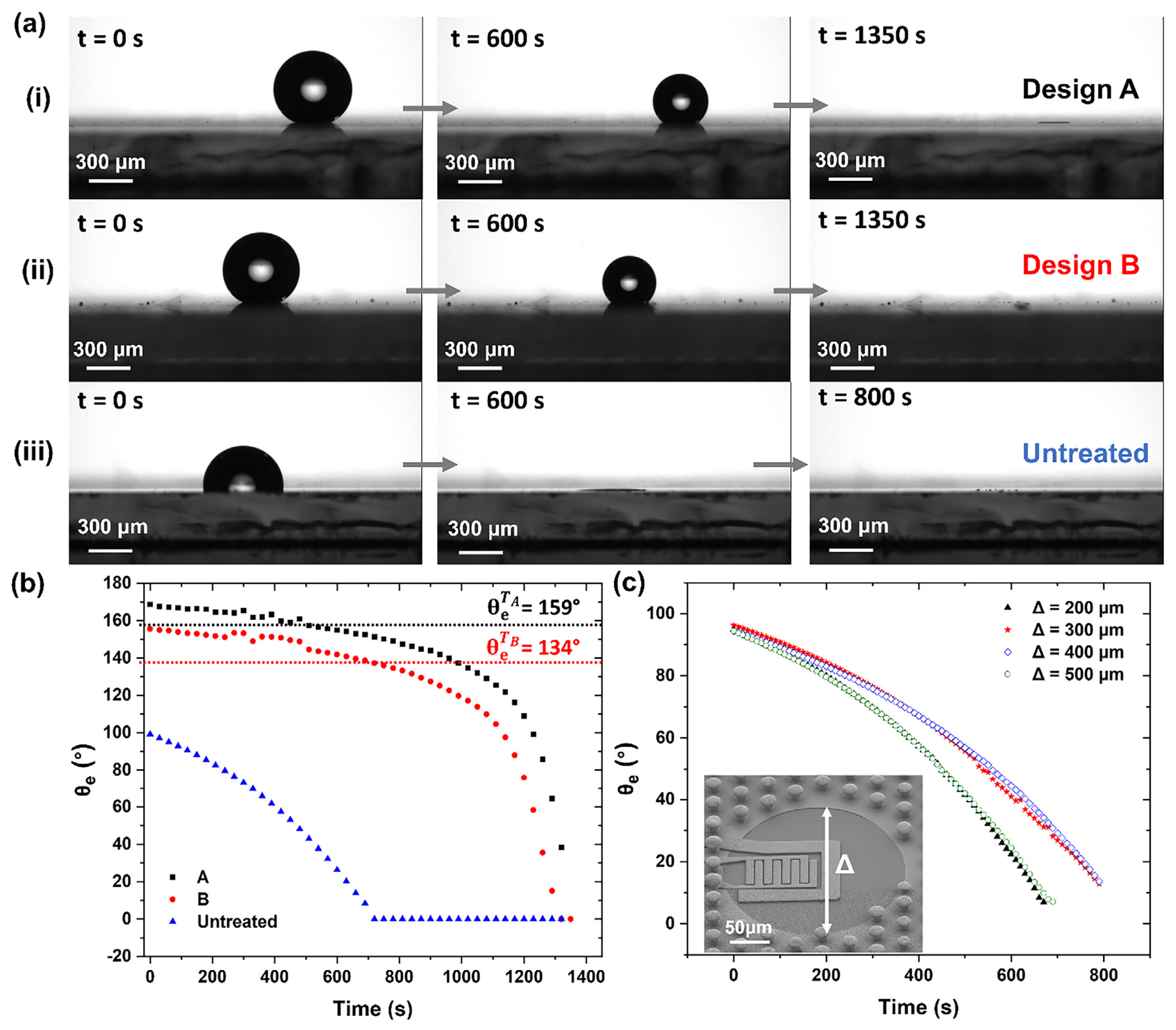
Evaluation of Wetting Behavior and Droplet Confinement in Micropillar Designs. (a) Time-lapse images showing evaporation dynamics for three surface types: (i) Design A (optimal SHP micropillar configuration), (ii) Design B (denser spacing), and (iii) untreated flat surface. (b) Apparent contact angle, $\theta_{e}$ vs. time for the three surface types, with black squares (Design A) and red circles (Design B); black and red dashed lines indicate the Cassie–Wenzel transition thresholds ($\theta_{e}^{T}$) of 159° and 134°, respectively.(c) Contact angle decay over time for four Δ values (200 – 500μm), showing minimal influence on self-centering behavior. Inset SEM shows the micropillar boundary (Δ) relative to the unstructured sensing area.

Here, d is the pillar diameter δ and the interpillar spacing as illustrated in Figure 1a (iii). Using this model, we identified an optimal geometry with d=7μm and δ=60μm, resulting in $\phi_{s}\approx 0.00989$ and a theoretical contact angle of $\theta_{e}\approx 170^{{}^{\circ}}$, closely matching our experimental measurements (Figure 2a (i) at t=0s and Figure 2b referred to as Design A). The base surface 3-aminopropyl) triethoxysilan (APTES)-treated planar Cr/W seed layer without any texture, exhibited $\theta_{0}\approx 100^{{}^{\circ}}$ (Figure 2a (iii) at t=0s and Figure 2b for the untreated surface), which we used to define the intrinsic wettability. However, as shown in Figure 2b for the untreated surface, in the absence of microstructures, the droplet spread significantly, underscoring the importance of the engineered texture in achieving hydrophobicity. Notably, we also evaluated the droplet behavior in the Wenzel regime [69], where the apparent contact angle is instead given by:

(5) $$cos\theta_{e}=rcos\theta_{0}$$

with r defined as the solid roughness factor, which is the ratio between the real surface and the projected one [70]:

(6) $$r=\frac{real\;(wetted)\;surface\;area}{projected\;(planform)\;area}$$

We can split the real area into base and sidewalls. However, the pillar tops just replace the same amount of base area; they do not add net area. Thus,

(7) $$r=1+\frac{sidewall\;area\;per\;unit\;cell}{projected\;area\;per\;unit\;cell}$$

With πdh as the sidewall area and $\frac{\sqrt{3}}{2}{\left(d+\delta \right)}^{2}$ as the unit-cell area for a hexagonal lattice, we will have:

(8) $$r=1+\frac{2\pi dh}{\sqrt{3}{\left(d+\delta \right)}^{2}}$$

where h (as shown in Figure 1a (i)) is the pillar height. To prevent the transition from Cassie–Baxter to Wenzel state during evaporation, we evaluated the Cassie–Wenzel transition threshold [71]:

(9) $$cos\theta_{e}^{T}=\frac{\phi_{s}-1}{r-\phi_{s}}$$

For our geometry, the solid roughness is r≈1.0679. With $\phi_{s}\approx 0.00989$, the transition threshold would be $\theta_{e}^{T}\approx 159^{{}^{\circ}}$. Since our experimentally measured angle (~170°) remains well above this threshold, the droplet consistently stays in the Cassie–Baxter regime as long as it is in the SHP region.

In addition to Design A, we evaluated another pillar design, Design B, maintaining the same pillar diameter (d) but reducing the interpillar spacing (δ) to assess its impact on key wetting characteristics. Both design parameters are calculated and summarized in Table 1. Additionally, a reference flat substrate without microstructures was tested to provide a baseline for comparison. All theoretical parameters for Design A, including solid fraction ($\phi_{s}$), surface roughness (r), and Cassie–Baxter-to-Wenzel transition thresholds ($\theta_{e}^{T}$), were previously derived and now serve as reference benchmarks. Time-lapse snapshots of the evaporation behavior for these three surfaces, Design A, Design B, and the untreated flat surface, are shown in Figure 2a, with corresponding contact angle measurements plotted in Figure 2b. Videos S1-S3 correspond to Designs A, B, and the untreated surface, respectively, and visualize droplet evolution over time. It is worth noting that the wet area includes APTES-treated Ni sidewalls, Ni tops, and exposed APTES-treated Cr/W base. The area-weighted intrinsic angle is $\approx 100^{{}^{\circ}}$, with negligible change to the Wenzel prediction.

As shown in Figure 2a (i), Design A maintained a spherical droplet shape during evaporation, indicating a stable Cassie–Baxter state. Design B, with denser pillars, showed a reduced contact angle and earlier collapse, suggesting a partial transition (Figure 2a (ii)). The untreated surface exhibited complete spreading and early collapse due to the lack of micropillarinduced confinement (Figure 2a (iii)). In Figure 2b, the wetting behavior differs between the two designs, and we explicitly verify whether full or partial transitions happen. For Design A, the measured contact angle stays well above the theoretical Cassie–Wenzel threshold (${\theta_{e}}^{T}\approx 159^{{}^{\circ}}$), starting at ~170° and gradually decreasing to ~160° by the end of evaporation. This confirms stable Cassie-Baxter behavior with no transition within the measurement window. For Design B, the apparent angle drops below its threshold (${\theta_{e}}^{T}\approx 134^{{}^{\circ}}$) after ~500 s; however, this decrease does not indicate to a full Wenzel transition. Instead, it reflects localized partial penetration of the meniscus into a small number of interpillar gaps near pillar edges during latestage evaporation, while most of the droplet remains suspended on trapped air. This align with its final apparent angle (~140°) and a moderate hysteresis of 10–20° [72]. Late-stage evaporation naturally increases the curvature of the liquid–vapor interface near pinned edge regions, raising the Laplace pressure within local meniscus pockets [73]; this rise in ΔP encourages partial, spatially confined penetration into interpillar gaps without causing global impalement. Similar Laplace-pressure-driven deformation and localized wetting transitions have been reported on superhydrophobic structures, where the curvature imbalance between the lower and upper menisci generates internal pressure gradients that can drive upward or lateral motion or induce limited wetting transitions in otherwise Cassie-stable droplets [74]. In our system, the effect is modest and only appears near the end of evaporation, consistent with the small (~10–20°) hysteresis and the final angle remaining well above a Wenzel state. Therefore, no full Wenzel transition was observed in either design during the evaporation prcocess; Design B exhibits only a brief, localized, metastable Cassie-to-partial-Wenzel transition driven by Laplace pressure and edge pinning, while the droplet mostly retains its Cassie-like character throughout evaporation, in line with evaporation-triggered partial wetting transitions reported in the literature [74].

This behavior matches evaporation-induced wetting dynamics reported in the literature, where partial penetration happens without full collapse, especially on microstructures with moderate roughness during the evaporation of small volumes. Hysteresis values of 10–20° are considered acceptable for sustaining functional superhydrophobicity [72].

Importantly, the selection of d=7μm and h=12μm was guided not only by theoretical models but also by fabrication feasibility. To implement this design through a rapid, scalable process with good manufacturing yield, we employed nickel electroplating with photoresist molds. This setup ensured uniform pillar growth with minimal fabrication defects, enabling high-throughput chip-scale integration. The entire microfabrication process, starting from E2RC base device preparation, followed by dielectric isolation, multilayer lithography, dry etching, and final electroplating, is outlined in the next section (Section 2.2).

### 2.2 ∣ Integration of the SHP Surface with the Sensing Device

One of the main challenges in manufacturing the SW-E2RC involves integrating two high–aspect-ratio structures – micropillars and 3D sensing microelectrodes – both fabricated through electrodeposition. Our developed fabrication process enables reliable formation of the micropillar and sensing layers while minimizing leakage between them. The fabrication of the core E2RC device (the sensing region, Figure 1b (i)) follows our previously established nanolithography-free protocol [39], which combines standard photolithography with template-driven nickel (Ni) electrodeposition. Briefly, interdigitated 3D microelectrodes are formed on glass substrates coated with a Cr/W seed layer. A two-layer positive photoresist defines the mold geometry, and electrodeposition is performed beyond the resist height to creat mushroom-like cap structures with controllable sub-micrometer gaps. After electrodeposition, the photoresist is stripped, and the Cr/W seed layer is selectively etched to isolate the electrodes. Following device fabrication, aluminum oxide (Al_2_O_3_) is deposited to electrically separate the sensing electrodes from the SHP micropillars (Figure 1b (ii)). Since Al_2_O_3_ is sensitive to the alkaline developers used in photolithography, we sputtered a Cr/W bilayer on top of the dielectric to serve as an etch-resistant hard mask for lithographic patterning (Figure 1b (iii)). Im, portantly, this Cr/W bilayer is also necessary as the conductive seed layer for electroplating the Ni micropillars. Therefore, the Al_2_O_3_ layer plays a critical role in maintaining electrical isolation between the E2RC electrodes and the Cr/W seed used for micropillar fabrication.

Before fabricating the micropillars for the SHP surface, it was essential to ensure that no pillars formed on the sensing region or electrical contact pads. To achieve this, we first spin-coated two layers of photoresist to reach the desired thickness (~3.5 μm in this work similar to the first generation of E2RC previously developed by our group) [32,]. Photolithography was then performed to define openings over the sensing area and contact pads (Figure 1b (iv) and Figure S1). Subsequently, using inductively coupled plasma reactive ion etching we sequentially removed the bimetal seed layer and the underlying Al_2_O_3_ in the exposed regions. This process exposed the sensing area and contact pads while preserving Al_2_ O_3_ elsewhere for electrical isolation (Figure 1b (v)). The etching depth and uniformity were confirmed using ellipsometry. For pillar electroplating, a polymeric layer was spin-coated and patterned to create cylindrical molds for nickel deposition (Figure 1b (vi)), with the resulting pillars having a height of 12 μm and diameter of 7 μm (Figure 1b (vii)), consistent with the geometries optimized to maintain the Cassie–Baxter wetting state during droplet evaporation. In some cases, during electrodeposition, the inter-pillar spacing became smaller than intended, and in certain regions, over-deposition beyond the resist thickness produced shortened pillars. These irregularities could compromise the hydrophobic performance of the surface, highlighting the importance of precise control in the pillar electrodeposition process. Figure 1c (i) shows SEM images of the pillars integrated with the sensing electrodes. Figure 1 (cii) presents a magnified view of the sensing area, displaying the generator and collector electrodes of the E2RC sensing microelectrodes. As in our previous work, we continued to use an on-chip reference/counter electrode, which had been previously validated for stable and reproducible performance. The inset in Figure 1c (iii) confirms successful fabrication of the pillars with a height of ~12μm, as expected based on the calculations discussed in Section 2.1. After completion of micropillar electroplating and photoresist stripping, the devices were placed in a vacuum desiccator containing 1mL of APTES and exposed to APTES vapor under reduced pressure (~100 mTorr) for 24 h at room-temperature to render both the Ni micropillars and the Cr/W seed layer uniformly hydrophobic with $\theta_{0}\approx 100^{{}^{\circ}}$ [75, 76].

To understand how the relative spacing of the sensing zone size with respect to the SHP area (Δ) affect droplet pinning, we fabricated devices with Δ=200μm, 300μm, 400μm, and 500μm, and tracked the contact angle evolution, as shown in Figure 2c. While the micropillar region exhibits a high apparent contact angle ($\theta_{e}\approx 170$), the central sensing zone was intentionally left unstructured, without micropillars, to allow direct electrochemical access for redox cycling. This untreated region lacks surface roughness required for superhydrophobicity and shows increased wettability, with a measured contact angle of ~100° (APTES-treated Ni), consistent with a smooth, hydrophilic surface. Consequently, the overall apparent contact angle of the SW-E2RC surface is reduced compared to the micropillar-only region (Figure S2). Although superhydrophobic surfaces are typically valued for preventing lateral spreading, studies by Lafuma and Quéré have shown that these surfaces can lose their repellency and transition to a Wenzel state when their texture becomes locally infiltrated [77]. Building on this, Mannetje et al. demonstrated that even micron-scale hydrophilic defects on a superhydrophobic surface can strongly pin and trap droplets, provided the trapping force of the defect exceeds any external force such as gravity or vibration. Their results indicate that, in the absence of strong external disturbances, the minimum defect area needed for pinning can be much smaller than the droplet’s contact area [78], validating our strategy of using small, well-defined hydrophilic regions to achieve reproducible droplet confinement and analyte enrichment in SW-E2RC devices with Δ as small as 200 μm.

When a droplet is dispensed onto the SW-E2RC surface, the superhydrophobic region strongly repels it due to its high contact angle and low adhesion. As the droplet moves forward, it is deflected by the surrounding SHP landscape and ultimately captured and pinned by the hydrophilic sensing zone, forming a self-aligned and consistently centered configuration. This behavior is demonstrated in Video S4, which first shows a droplet on an untreated smooth surface, followed by its rapid repulsion from the SHP region, and finally its stable anchoring on the unstructured sensing zone, where it remains confined throughout evaporation. Additionally, Videos S5-S8 show that the droplet consistently self-centers on the sensing area regardless of Δ. Contact angle measurements and visual analysis further confirmed that this space variation had minimal impact on droplet shape or placement, indicating that the self-alignment effect is robust across all tested configurations. Without superhydrophobic confinement, the droplet spreads freely and adheres randomly wherever it is manually dispensed, with no specific localization, emphasizing the importance of the SW-E2RC design in ensuring consistent droplet confinement and reproducibility. This passive centering mechanism not only improves reproducibility but also enhances detection sensitivity by ensuring uniform evaporation dynamics and maximizing interaction between analyte molecules and the sensor surface. Unlike the first generation E2RC, where users had to manually align droplets, SW-E2RC achieves robust and automatic droplet localization without external aids. The results in Section 2.4 demonstrate improved sensitivity of SW-E2RC devices compared to E2RC devices.

### 2.3 ∣ Numerical Analysis of the SW-E2RC Devices

Before discussing the experimental results with SW-E2RC for virus detection, we performed finite element analysis (FEA) simulations for two coupled mechanisms: (i) evaporation-driven enrichment of the redox couple, and (ii) selective-wettability-controlled droplet localization, which stabilizes RC currents in SW-E2RC. We developed a time-dependent model in COMSOL Multiphysics to better understand the parameters involved in the electrochemical system in an evaporating droplet, and explicitly focusing on the current evolution through redox cycling. The parameters used in building the COMSOL model are summarized in Table 2.

**FIGURE 3:**
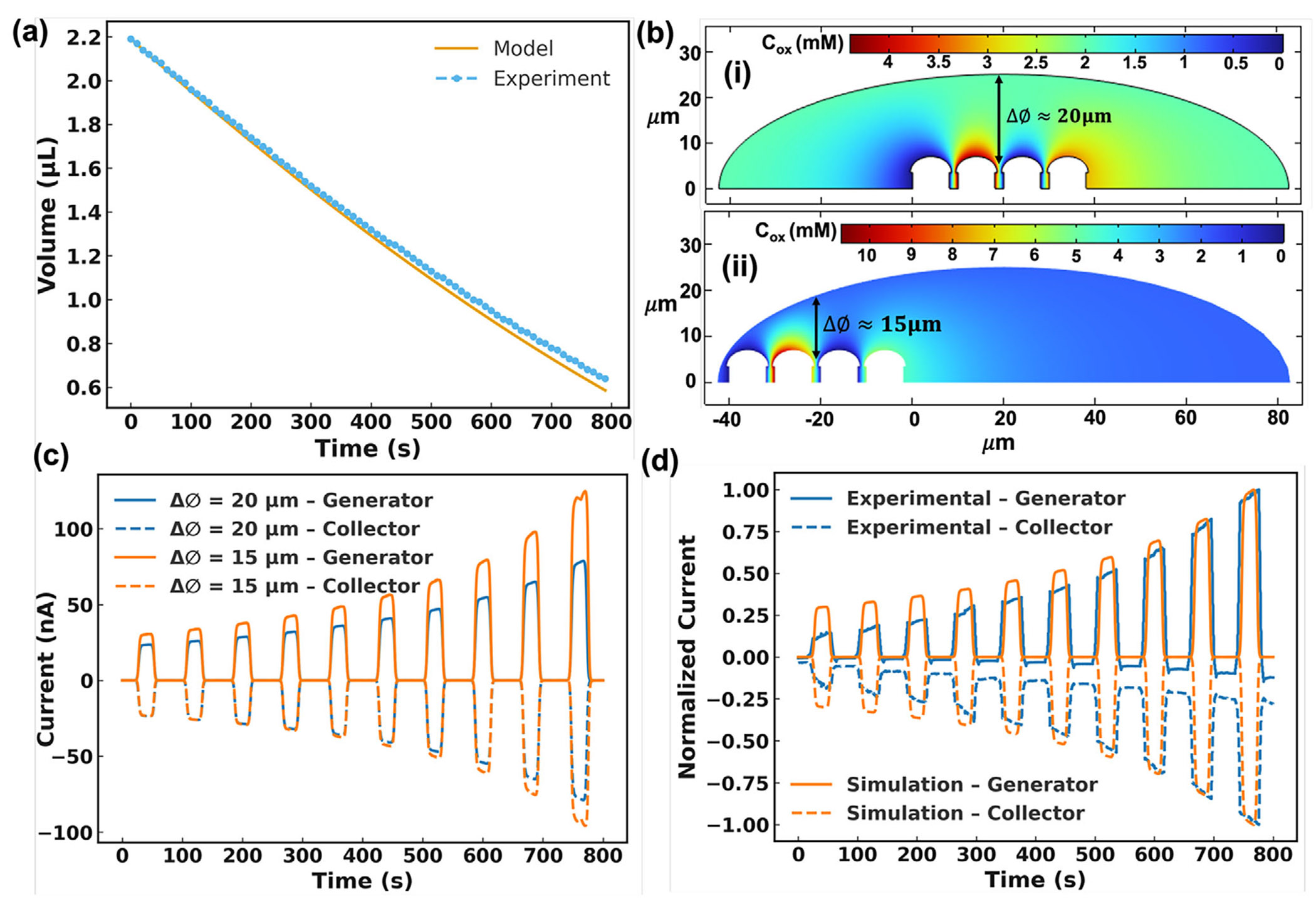
Analytical and numerical analysis of simultanous droplet evaporation and redox cycling in SW-E2RC. (a) Comparison of experimental droplet volume with the power-law decay model, showing strong agreement over time. (b) Schematic of lateral offset (ΔØ) between the droplet’s enriched boundary and the generator-collector electrodes, used to parameterize placement sensitivity. (c) Simulated generator and collector currents for two droplet positions (ΔØ≈15μm and 20μm) over ten cycles. The current varies with the droplet location, confirming the role of selective wettability (which stabilizes droplet positioning) on detection accuracy. (d) Comparison of normalized peak currents from simulation and experiment across cycles, both showing signal amplification that tracks the theoretical concentration enrichment (resulting from evaporation) predicted by Equation (11) (and the plots in part a).

To model droplet evaporation and volume change over time (which is necessary for the COMSOL simulations), we adopted a power-law decay function for the droplet volume as developed by Dak et al. [39]. The evaporating sessile droplet was assumed to undergo a quasi-static shape transformation, maintaining a pinned contact line. The droplet volume V(t) was expressed as:

(10) $$V(t)=V_{0}{\left(1-\frac{t}{t_{f}}\right)}^{n}$$

where $V_{0}$ is the initial droplet volume (1.5 μL), $t_{f}$ is the total evaporation time (~ 1200 s), n and is the exponent fitted from experimental droplet volume data (found to be ~1.5). As the droplet volume decreased, the concentration of the redox molecules (${\left[Fe{\left(CN\right)}_{6}\right]}^{4-}$) increased accordingly. Figure 3a shows a good correlation of the droplet in experimental data (measured with a contact angle instrument) with the dynamic droplet volume in Equation (10). Assuming no loss of analyte due to adsorption or reaction beyond redox cycling, the time-dependent concentration C(t) was derived from mass conservation:

(11) $$C(t)=\frac{C_{0}V_{0}}{V(t)}=\frac{C_{0}}{{\left(1-\frac{t}{t_{f}}\right)}^{n}}$$

where $C_{0}$ is the initial concentration of ${\left[Fe{\left(CN\right)}_{6}\right]}^{4-}$. Figure S3b (i) shows the C(t) increasing over time. In the COM-SOL simulations, analytically-derived C(t) – using Equation (11) – was applied at the droplet-electrolyte boundary to drive the redox reactions (Figure S3b (ii)), thus enabling simulation of the evaporation-enhanced signal amplification observed experimentally.

Before the experimental demonstration of the importance of droplet placement on sensitivity (Section 2.4), we conducted a simulation study: we parameterized droplet position by the lateral offset ΔØ between the droplet’s enriched boundary and the electrode tips (Figure 3b). We set ΔØ≈15μm or 20μm by shifting the enriched boundary field relative to the electrodes, while keeping V(t) constant across cases. The current-vs-cycle results (Figure 3c) show that droplets with a shorter lateral offset (ΔØ≈15μm) indicating a misaligned but closer position to the electrodes -produce higher generator and collector currents due to a shorter diffusion path. Conversely, droplets with a larger offset (ΔØ≈20μm), representing centered and aligned placement, yield lower currents due to the increased diffusion distance. These results support our hypotheses: selective wettability ensures that droplets are consistently pinned at the center of the sensing zone's center, stabilizing both redox concentration and diffusion geometry to reduce signal variability. To evaluate how well the simulated evaporation-driven signal amplification aligns with the experimental data, we plotted the normalized peak currents over successive cycles in Figure 3d. Both simulation and experiment exhibit consistent current amplification that closely follows the theoretical inverse volume trend $C_{0}∕C(t)$, as described in Equation (11). This correlation confirms that droplet evaporation is the main factor driving current amplification in SW-E2RC.

### 2.4 ∣ Validating the Performance of SW-E2RC with mNP-Captured Viral Particles

To validate the analytical performance of SW-E2RC, we tested inactivated virons of SARS-CoV-2 (Omicron variant) and H5N1. Before adding droplets to count viral particles, we first captured the target virus using magnetic nanoparticles (mNPs) functionalized with virus-specific antibodies. After incubating with mNPs, we performed magnetic separation and thermal elution to isolate the mNPs from the viral particles following separation from the initial sample. The thermal elution improves sensitivity by reducing the noise caused by unbound mNPs. For SARS-CoV-2, the initial stock had a concentration of 1.16 × 10^9^ copies/mL (validated with RT-PCR). Serial dilution was performed to prepare samples for analysis with the sensors and RT-PCR. The RT-PCR Ct values increased with dilution, indicating a decrease in viral genome copies. RT-PCR targeting the influenza M gene (M+64) showed strong amplification at higher concentrations, with Ct values rising systematically across the series.

**FIGURE 4:**
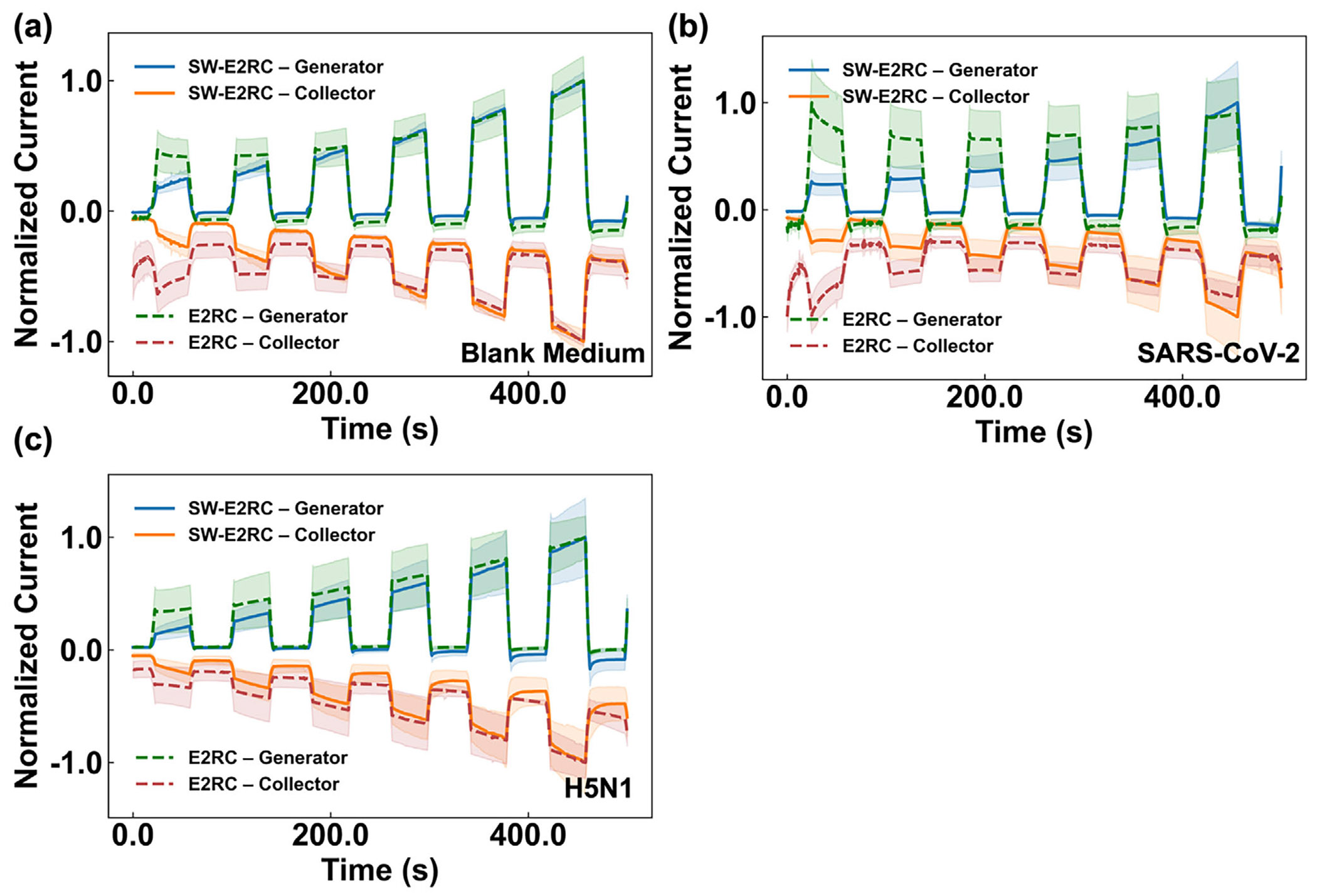
Comparison of redox-cycling currents in SW-E2RC vs. E2RC: (a) blank medium, (b) SARS-CoV-2 (7.28 × 10^7^ copies/mL), and (c) H5N1 (7.28 × 10^7^ copies/mL). SW-E2RC utilizes droplet evaporation, redox cycling, and selective wettability to confine droplets on a hydrophilic sensing zone, improving mass transport and cycling efficiency. Compared to E2RC, SW-E2RC produces less noise and maintains more consistent generator-collector symmetry (higher collection efficiency), allowing for more sensitive and reproducible detection at ultralow viral concentrations. Data are presented as mean ± SD (*n* = 3).

To understand the effect of selective wetting on the real-time signal, we first compared the time-evolution of the redox cycling signals using SW-E2RC and E2RC. Figure 4 shows the average generator and collector peak currents (normalized to the highest mean current for generator and collector, respectively) with blank medium (the base electrolyte solution without any target analyte) and virion-containing droplets (7.28 × 10^7^ copies/mL for SARS-CoV-2 and H5N1). The current profiles indicate that incorporating selective wettability into the E2RC design consistently produces higher generator and collector currents under all tested conditions (Figure S3). For both viruses (SARS-CoV-2 and H5N1), SW-E2RC shows a steady increase in generator and collector currents over time (Figure 4b; Figure S4a,c) while E2RC shows lower amplitudes and less signal change over time (indicating reduced sensitivity); Figure 4c and Figure S4b,d.

Next, we compared the analytical performance of SW-E2RC with E2RC in response to viral particles by defining the signal as ΔS (similar to our previous work [32]):

(12) ΔS≡S(i)−S(f)

**FIGURE 5:**
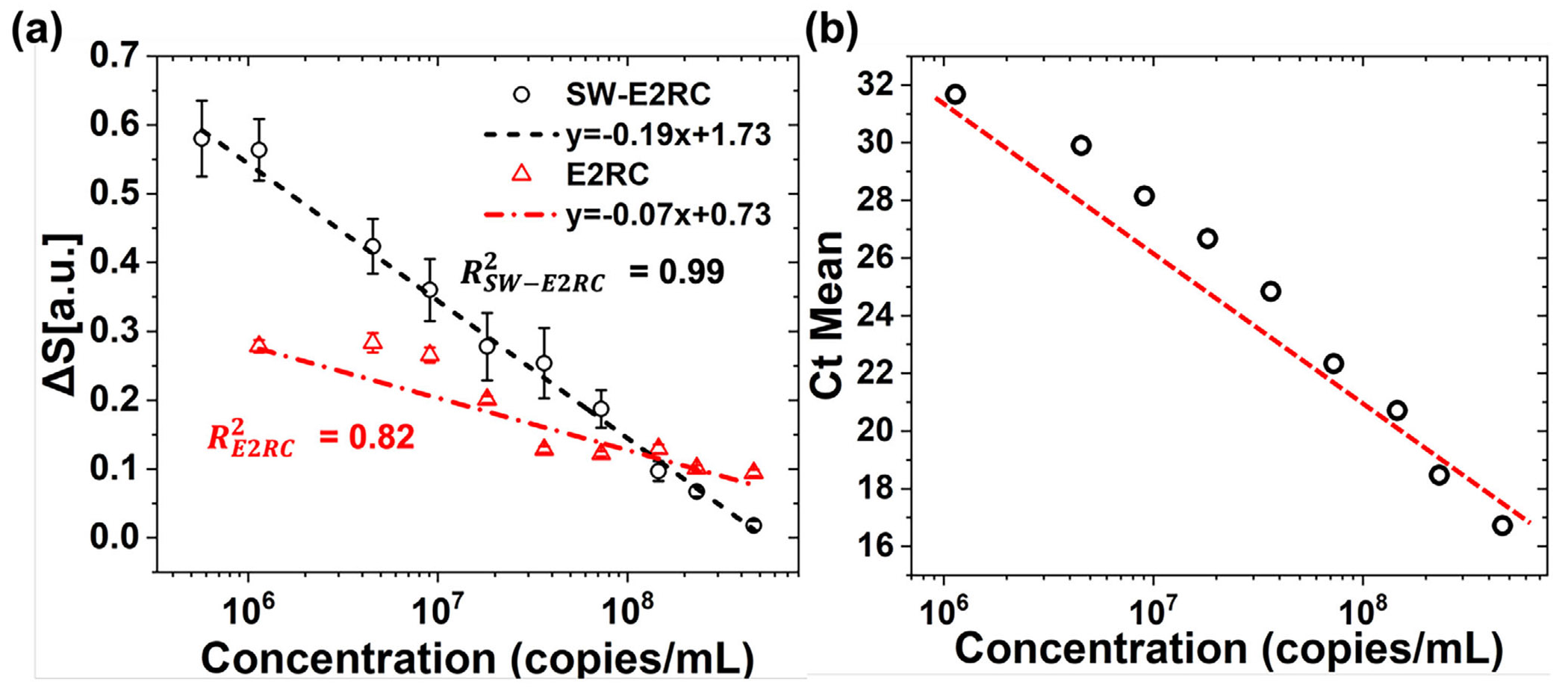
Quantitative comparison of ΔS between SW-E2RC and E2RC platforms for detection of SARS-CoV-2 across a clinical dilution series. (a) ΔS is defined as the linear change in the normalized generator current over nine redox cycling sweeps Equation (12). SW-E2RC offers significantly higher linearity and a lower LOD than E2RC. Data shown as mean ±SE(n=9). (b) Corresponding RT-PCR cycle threshold (Ct) values confirm a strong correlation between ΔS and viral genome abundance.

Where $S\equiv I_{generator,blank\;medium}∕I_{generator,p}$. Here, $I_{generator,blank\;medium}$ refers to the generator peak current for the blank medium, and $I_{generator,\rho }$ refers to the generator peak current in each RC cycle for the virus-containing droplet. S(i) and S(f) denote the initial and final values of S during redox cycling, obtained at the beginning and end of the measurement window, respectively. ΔS was determined by linear fitting of the S across nine RC cycles. To provide a clearer context, ΔS varies based on (i) the number of analyzed RC cycles and (ii) the particle-size regime. As demonstrated in our previous work [32], ΔS values during the first ~5 cycles are dominated by transient interfacial effects and only stabilize after evaporation-driven enrichment begins. Additionally, the particle-size-dependent behavior of ΔS arises because smaller nanoparticles, comparable to the IDE gap scale, produce stronger and more stable ΔS signatures because they more effectively permeate the electric field and access the redox-cycling interface. Figure 5a plots ΔS of SW-E2RC and E2RC across different concentrations of SARS-CoV-2, while corresponding RT-PCR Ct values are shown in Figure 5b. Both detection platforms showed decreasing ΔS values as Ct decreased (which corresponds to higher viral concentration), confirming a strong link between the electrochemical signal and viral genome levels. Notably, SW-E2RC exhibited higher ΔS values across all concentrations, particularly at the lower end of the detection range, indicating greater sensitivity and a wider dynamic range compared to E2RC.

The calibration curve for SW-E2RC (Figure 5a) exhibited excellent linearity, with a regression coefficient of $R^{2}=0.99$, while E2RC showed a weaker fit ($R^{2}=0.82$). Samples that did not produce a Ct value in RT-PCR were used as blanks in electrochemical measurements to determine the LOD values. For SW-E2RC (with $\Delta S_{blank}=0.77$ and σ=0.25), the LOD was calculated as 9.2 × 10^3^ copies/mL, where σ is the standard deviation of the blank and m is the slope of the calibration curve in the linear-logarithmic scale. For E2RC (with $\Delta S_{blank}=0.27$ and σ=0.027), the LOD was estimated as 4.11 × 10^7^ copies/mL, which is at least three orders of magnitude worse than SW-E2RC. Besides showing better sensitivity and linearity, SW-E2RC demonstrated a broader dynamic concentration range compared to E2RC. These results further confirm the enhanced detection performance of SW-E2RC, attributed to its integrated selective-wettability-based droplet confinement.

### 2.5 ∣ Effect of Particle Size and Surface Charge on Detection Sensitivity

**FIGURE 6:**
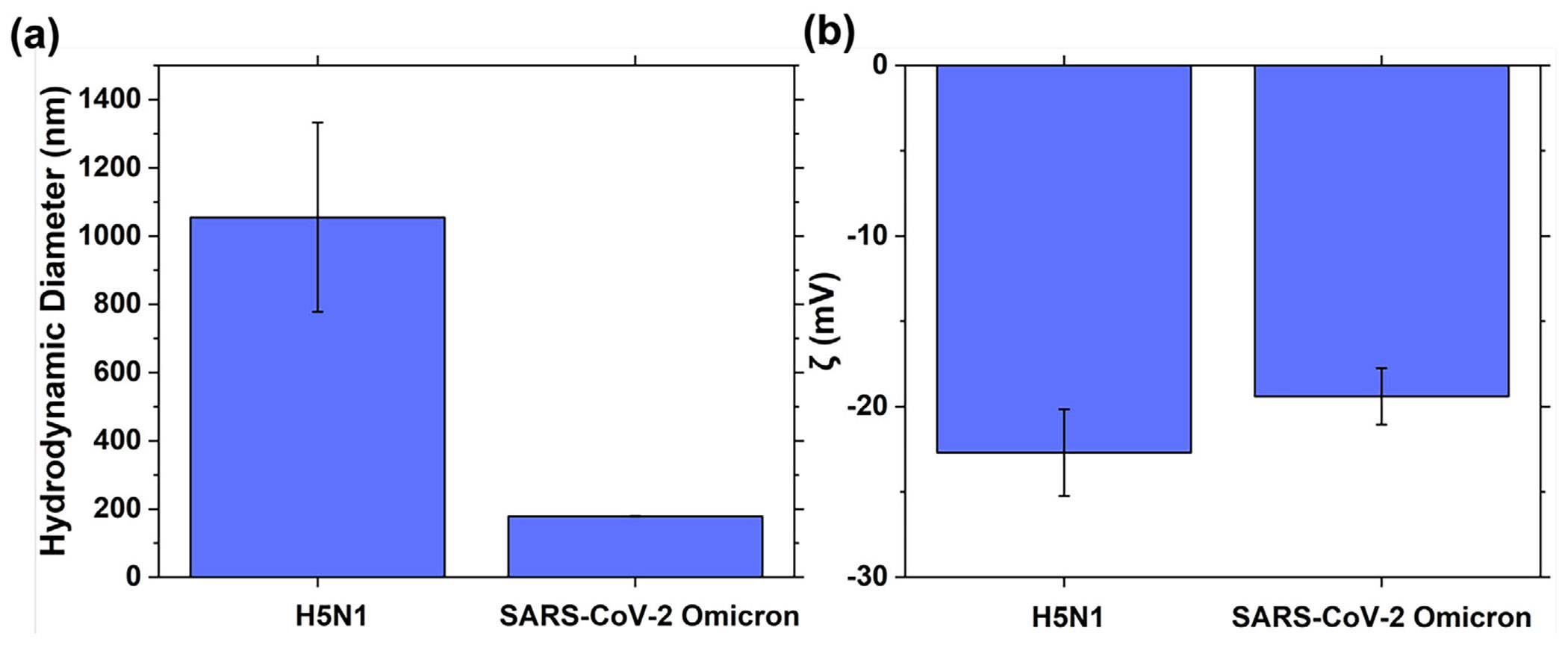
Physical characterization of SARS-CoV-2 and H5N1 virions. (a) Dynamic light scattering (DLS) measurements show that H5N1 has a larger hydrodynamic diameter (> 1000 nm) compared to SARS-CoV-2 (∼180nm). (b) Zeta potential analysis indicates similar negative surface charges (≈−20mV) for both viruses. The notable size difference, rather than charge differences, likely explains the reduced ΔS response observed for H5N1 in SW-E2RC and E2RC devices, consistent with our prior study with different sized beads demonstrating steric exclusion effects in confined electrode geometries. Data are presented as mean ± SD (*n* = 3).

In addition to SARS-CoV-2, the SW-E2RC platform was tested for H5N1 virus detection to demonstrate broader applicability (Figure S5). Similar to the response to SARS-CoV-2, ΔS decreases with the increase of H5N1, as the H5N1 concentration increases. Notably though, we observed a greater variability and less sensitivity when comparing the SW-E2RC response to H5N1 compared to SARS-CoV-2. We hypothesized that the weaker response to H5N1 compared to SARS-CoV-2 is related to differences in their physical and surface charge properties. To test this hypothesis, we analyzed the hydrodynamic size and surface charge of SARS-CoV-2 and H5N1 viral particles using dynamic light scattering (DLS) and zeta potential (ζ) analysis, respectively (Figure 6a,b). Figure 6a shows that the average hydrodynamic diameter of H5N1 viral particles exceeded 1000 nm, significantly larger than that of SARS-Cov-2 (∼180nm). Conversely, zeta potential measurements (Figure 6b) revealed that both viruses had similar negative surface charges, indicating comparable electrophoretic mobility under the testing conditions. The similarity in ζ-potential implies that electrostatic attraction to the electrode surface is not the primary factor affecting detection; instead, the large size difference likely has the dominant effect. As detailed in Section 2.4, ΔS values for SARS-CoV-2 showed a clear, concentration-dependent suppression trend, while H5N1 responses were more inconsistent. We believe this difference istems from the geometric constraints of the E2RC device, which was originally designed for particles < 200 nm, like SARS-CoV-2. The much larger H5N1 virions have limited access to the sub-micrometer interdigitated gaps (~ 500 nm gap), decreasing their ability to cause surface blockage and thus weakening the signal. This interpretation aligns with well our earlier bead-size study, where 100 nm negatively charged particles produced significantly higher ΔS responses than 1 μm beads, regardless of surface charge polarity [32]. In that study, the decreased interaction of larger beads with the sensing surface was attributed to steric exclusion from the confined electrode regions, a mechanism similar to the reduced response observed here for H5N1 virus. These results show that while surface charge can affect electrophoretic attraction in many biosensing scenarios, particle size is the main factor affecting detection sensitivity in our current setup. This emphasizes the importance of designing electrode shapes to be close to the target analyte’s size when adapting SW-E2RC technology to detect viruses, bacteria, or other biological targets with different physical sizes.

## 3 ∣ Conclusion

In this study, we developed and validated the SW-E2RC biosensing system, which combines selective wettability with evaporation-enhanced redox cycling. By integrating a hydrophilic sensing zone within a superhydrophobic micropillar lattice, the system enables precise droplet confinement, increased analyte enrichment through evaporation, and reproducible self-alignment without manual intervention. Manufactured using a scalable and nanofabrication-free process, the SW-E2RC platform outperforms the first generation E2RC devices, with ~10^3^-times better LOD, higher sensitivity, improved linearity, and a wider dynamic range. Validation with SARS-CoV-2 (size of < 200 nm) and H5N1 (size of ~1 μm) viruses revealed a size-dependent response linked to the sub-micrometer sensing gap, with optimal sensitivity for particles closer in size to the sensor’s critical dimension (mainly the gap between generator and collector electrodes). Dynamic light scattering and Zeta potential measurements confirmed similar negative surface charges for both viruses, indicating that size-driven steric accessibility primarily influenced sensitivity differences. These findings highlight the potential of SW-E2RC as a robust, tunable, and field-ready diagnostic platform capable of sensitive pathogen detection in microliter-scale samples. Beyond this specific application, the principle of selective-wettability can be broadly applied to other microfluidic and biosensing platforms. Controlled surface heterogeneity can be utilized for precise droplet manipulation, enhanced analyte pre-concentration, and improved redox-cycling efficiency across various biosensor architectures, emphasizing the broader relevance and adaptability of the SW-E2RC concept for next-generation diagnostic devices.

## 4 ∣ Methods

### 4.1 ∣ Materials and Reagents

Nickel electroplating solution (Nickel Sulfamate, Transene Company Inc., USA) was used for substrate coating and electrode fabrication. Positive photoresist (SPR955, JSR Corporation, USA) and AZ4620 photoresist (MicroChemicals, Germany) were used for lithography and mold patterning. Aluminum oxide (Al_2_O_3_) was deposited by atomic layer deposition (ALD) using trimethylaluminum (TMA) and deionized water as precursors (Cambridge NanoTech, USA). Potassium hexacyanoferrate (II) trihydrate (K_4_[Fe(CN)_6_]·3H_2_O, Sigma–Aldrich, USA) and potassium chloride (KCl, Sigma–Aldrich, USA) were used as the redox probe and supporting electrolyte, respectively. 3-aminopropyl) triethoxysilan (APTES, Sigma Alderich, USA) was used to render both the Ni micropillars and the Cr/W seed layer uniformly hydrophobicity. Heat-inactivated SARS-CoV-2 (Isolate hCoV-19/USA/GA-EHC-2811C/2021, Lineage B.1.1.529; Omicron Variant, Gamma-Irradiated, NR-56496) and Influenza A Virus (A/mallard/Wisconsin/2576/2009, H5N1, Gamma-Irradiated, NR-59421) were obtained through BEI Resources, NIAID, NIH. The antibody used for SARS-CoV-2 Omicron was a rabbit polyclonal anti-Spike S2 antibody (Sino Biological, China; Product #: 40590-T62). The following reagent was obtained through BEI Resources, NIAID, NIH: Monoclonal Anti-Influenza Virus H5 Hemagglutinin (HA) Protein (VN04-8), A/Vietnam/1203/2004 (H5N1), (ascites, Mouse), NR-2733. Magnetic nanoparticles coated with Protein A (80 nm BNF-Starch, Micromod, Germany) were used for magnetic virus capture and thermal elution. PBST buffer (1 × PBS + 0.1% Tween-20, Sigma–Aldrich, USA) and standard PBS (pH 7.4) were prepared using analytical-grade reagents, and all dilutions and sample preparations were performed using ultrapure deionized water (18.2 MΩ·cm, Milli-Q, MilliporeSigma, USA).

### 4.2 ∣ Fabrication of Superhydrophobic-E2RC Devices with Electroplated Pillars

Device fabrication steps, including electrode definition, dielectric passivation, selective etching, and micropillar formation, were performed in the Penn State Nanofabrication Laboratory. Photolithography was carried out using a maskless aligner (MLA150, Heidelberg Instruments, Germany). Nickel electroplating was performed with a two-electrode setup powered by a PalmSens4 potentiostat (PalmSens BV, USA), with an iron plate serving as the counter electrode. A constant current density of 20 mA/cm^2^ was applied for 900 s, resulting in the deposition of ~12 μm tall Ni pillars. A 90 nm-thick aluminum oxide (Al_2_O_3_) dielectric layer was deposited using an ALD150LE system (Kurt J. Lesker Company, USA) at 200°C, employing trimethylaluminum (TMA) and water as precursors. Each ALD cycle included a 0.015 s TMA pulse, a 5 s N_2_ purge, a 0.015 s H_2_O pulse, and a 5 s N_2_ purge, repeated for 1000 cycles. Selective dry etching of the Cr/W bilayer and the underlying Al_2_O_3_ layer was performed using a Versalock 700 ICP-RIE system (SPTS Technologies, UK). Etching was carried out with 2 sccm Cl_2_ and 20 sccm BCl_3_ gas flow at a pressure of 10 mTorr and an RF power of 900 W. The etch rate was approximately 6 Å/s, ensuring controlled and uniform material removal.

### 4.3 ∣ Characterization of Sensors using SEM and Ellipsometry

To characterize the surface properties of the fabricated sensors, various characterization techniques were used. The morphology and elemental composition of the final sensor were examined with Scanning Electron Microscopy (SEM) (Carl Zeiss AG, Germany). Film thicknesses and etch depths were confirmed using spectroscopic ellipsometry (M-2000, J.A. Woollam, USA).

### 4.4 ∣ Surface Wettability Characterization

Contact angle measurements were performed to assess the wettability of the fabricated surfaces and to confirm the transition between Cassie–Baxter and Wenzel states. Static contact angles were measured using a contact angle goniometer (Model 260, Ramé-hart Instrument Co., USA) at room-temperature with 1.5 μL DI water droplets. Measurements were taken at multiple time points during droplet evaporation to analyze dynamic wetting behavior across different micropillar configurations.

### 4.5 ∣ Virus Sample Preparation and Thermal Elution

To selectively capture viral particles, magnetic nanoparticles (mNPs) coated with Protein A were functionalized with virus-specific antibodies. Specifically, 2.5 × 10^10^ mNPs were washed once using a magnetic stand and incubated with either anti-Spike S2 polyclonal antibody (for SARS-CoV-2) or monoclonal anti-H5 HA antibody (for H5N1) diluted 1:2000 in PBST buffer (1× PBS + 0.1% Tween-20). The antibody–mNP mixture was incubated at room-temperature for 60 min, followed by three washing steps using PBST (each for 3 h on a magnetic stand) to remove unbound antibodies. For viral capture, a defined concentration of the virus sample (1.16 × 10^9^ – 2.83 × 10^5^ copies/mL for SARS-CoV-2 and 1.16 × 10^9^ – 2.26 × 10^6^ copies/mL for H5N1) was directly added to the antibody-conjugated mNPs (Ab-mNPs) and incubated for 1 h at room-temperature. After conjugation, the mNP–virus complexes were magnetically separated and washed twice with PBS. Thermal elution was performed by resuspending the pellet in PBS and incubating the sample at 80°C for 3 h to release intact virions into the solution. After elution, the magnetic beads were removed, and the resulting supernatant, containing the eluted virus, was used for electrochemical detection. Final measurements were performed in 1 mm potassium ferrocyanide ([*Fe*(*CN*)_6_]^4−^) in 10 mm KCl, prepared using ultrapure deionized water. No further dilution was applied after elution; each sample corresponded to its defined virus concentration prior to thermal release and testing with SW-E2RC devices.

### 4.6 ∣ Measurement of Zeta Potential

Zeta potential (ζ) and hydrodynamic size measurements were carried out using a Zetasizer Nano ZS (Malvern Instruments Ltd., UK), which combines electrophoretic light scattering and dynamic light scattering (DLS) for accurate particle characterization. All measurements took place at 22°C in an 800 μL disposable folded capillary cell. The Zetasizer Software version 6.20 was used with the zeta potential and size measurement modes activated. Heat-inactivated SARS-CoV-2 and H5N1 virus samples prepared at the same concentration (5.8 × 10^8^ *copies/mL*), were washed three times with sterilized ultrapure deionized water (DIW) using an ultracentrifuge (Optima TLX 120K, Beckman Coulter, USA) at 80 000 × g for 1 h to remove residual storage media. After washing, both free virus samples and antibody-conjugated magnetic nanoparticle–virus complexes (Ab-mNP–virus) were resuspended in 1000 μL of sterilized DIW. A 120 s equilibration period was allowed before each measurment. Zeta potential and DLS readings were performed in auto mode with three replicates per sample, each rconsisting of 10–100 runs depending on signal stability. For DLS anlysis, measurements used a backscatter angle of 173°, and data were processed using a general-purpose analysis model with automatic attenuation and duration settings.

### 4.7 ∣ Electrochemical Measurements

A dual-channel CHI760E Potentiostat (CH Instruments, Inc., U.S.) was used to control the applied potential and measure the resulting current, allowing the collection of RC data. During redox cycling the generator electrode was set to sweep from −1 to 1 V at 50 mV/s, with a samples interval of 5 mV, while the collector electrode was biased at a constant potential of 1 V. For single-mode measurement, the potential was swept from −1 to 1 V at 50 mV/s; with a 5 mV sample interval, and the other electrode was left floating.

### 4.8 ∣ Reverse Transcription Polymerase Chain Reaction (RT-PCR)

RNA extraction and RT-PCR assays were carried out for Influenza A virus (H5N1) and SARS-CoV-2. Viral suspensions were serially diluted to prepare samples with varying genome concentrations expressed in copies/mL, and 200 μL from each dilution was processed for RNA extraction using the KingFisher Flex system (Thermo Fisher Scientific, Waltham, MA, USA) alonge with the MagMAX Viral/Pathogen Nucleic Acid Isolation Kit (Thermo Fisher Scientific, catalog no. A42352), following the manufacturer’s instructions. RT-PCR was performed on an ABI 7500 Fast instrument (Thermo Fisher Scientific) with the AgPath-ID One-Step RT-PCR reagents (Thermo Fisher Scientific, catalog no. AM1005). For H5N1 detection, primer-probe sets targeting the matrix (M) gene and the H5 gene were employed, including sequences reported by Spackman et al. [79]. M+25, AGA TGA GTC TTC TAA CCG AGG TCG; M−124, TGC AAA AAC ATC TTC AAG TCT CTG; and M+64 probe, FAM-TCA GGC CCC CTC AAA GCC GA-TAMRA. For SARS-CoV-2 detection, assays targeted the N1 gene using the RUO qPCR Primer & Probe Kit (Integrated DNA Technologies, catalog no. 10006713). Cycle threshold (Ct) values were automatically calculateded using the ABI 7500 system software.

### 4.9 ∣ Finite Element Analysis

Finite element analysis (FEA) was conducted using COM-SOL Multiphysics version 6.2, featuring the Tertiary Current Distribution (Nernst–Planck transport under electroneutrality) electrochemistry module with Butler–Volmer kinetics applied at the electrode boundaries. The geometry was modeled as 2D axisymmetric around the center of the sensing zone and included two coplanar working electrodes: a generator sweeping from −1.0 to + 1.0 V at 50 mV s^−1^ for ten cycles, and a collector held at −1.0 V (Figure S3a). Species transport for the oxidized (Ox) and reduced (Red) forms was described by the Nernst–Planck equation with no convection. Bulk electrolyte concentration, diffusion coefficients, and other kinetic parameters were taken from the literature and our previous study (Table 2) [32, 80].

As shown in Figure S3c, the computational domain was discretized with an unstructured triangular mesh, with local refinement used to capture steep gradients in potential and current density near electrode edges and within narrow gaps.

### 4.10 ∣ Statistical Analysis

The mean, standard deviation, and standard error of the redox cycling data were calculated based on at least three individual sensors (independently fabricated) and a minimum of three scans for each sensor. All statistical analyses were conducted using OriginPro software. The curve stats module in OriginPro software was utilized for redox cycling and ΔS analysis.

## Supplementary Material

SI

Video S8

Video S6

Video S7

Video S4

Video S5

Video S3

Video S2

Video S1

Additional supporting information can be found online in the Supporting Information section.

Supporting File 1: smtd70471-sup-0001-SuppMat.docx.

Supporting File 2: smtd70471-sup-0002-VideoS1.mp4.

Supporting File 3: smtd70471-sup-0003-VideoS2.mp4.

Supporting File 4: smtd70471-sup-0004-VideoS3.mp4.

Supporting File 5: smtd70471-sup-0005-VideoS4.mp4.

Supporting File 6: smtd70471-sup-0006-VideoS5.mp4.

Supporting File 7: smtd70471-sup-0007-VideoS6.mp4.

Supporting File 8: smtd70471-sup-0008-VideoS7.mp4.

Supporting File 9: smtd70471-sup-0009-VideoS8.mp4.

## Figures and Tables

**TABLE 1 ∣:** Geometric and theoretical wetting parameters for Designs A, B.

Design	d	δ	$\phi_{s}$	$\theta_{e}$	r	$\delta_{e}^{T}$
A	7 μm	60 μm	0.00989	~170°	1.0679	~159°
B	7 μm	20 μm	0.0609	~161°	1.418	~134°

**TABLE 2 ∣:** The electrochemical parameters used in the COMSOL simulations.

Parameter	Value
$C_{0}$	1 mm
$k^{0}$	0.1 cm s^−1^
$D_{ox}$	7.2 × 10^−6^ cm^2^ s^−1^
$D_{red}$	6.4 × 10^−6^ cm^2^ s^−1^
$a_{a}$	0.50
n	1
T	293 K

## Data Availability

The data that support the findings of this study are available from the corresponding author upon reasonable request.
