# Supplementary material for: Selective Wettability–Driven Evaporation-Enhanced Redox Cycling for Robust and Ultrasensitive Detection of Viral Particles
Source: Small Methods. Author manuscript; Available in PMC 2026 Mar 31. (PMC12929934; doi:10.1002/smtd.202502229)
Supplement: SI [file NIHMS2144757-supplement-SI.docx]

Supporting Information for

**Selective Wettability–Driven Evaporation-Enhanced Redox Cycling for Robust and Ultrasensitive Detection of Viral Particles**

# Pouya Soltan Khamsi^1,2^, Shubhada K Chothe^3,4^, Suresh V Kuchipudi^3,4^, and Aida Ebrahimi^1,2,5*^

^1^Department of Electrical Engineering, ^2^Materials Research Institute, The Pennsylvania State University, University Park, Pennsylvania 16802, United States

^3^Department of Infectious Diseases and Microbiology, School of Public Health, University of Pittsburgh, Pittsburgh, Pennsylvania 15260, United States

^4^Center for Vaccine Research, University of Pittsburgh, Pittsburgh, Pennsylvania, 15261, United States

^5^Department of Biomedical Engineering, The Pennsylvania State University, University Park, Pennsylvania 16802, United States

*Corresponding author: [sue66@psu.edu](mailto:sue66@psu.edu)

1. *
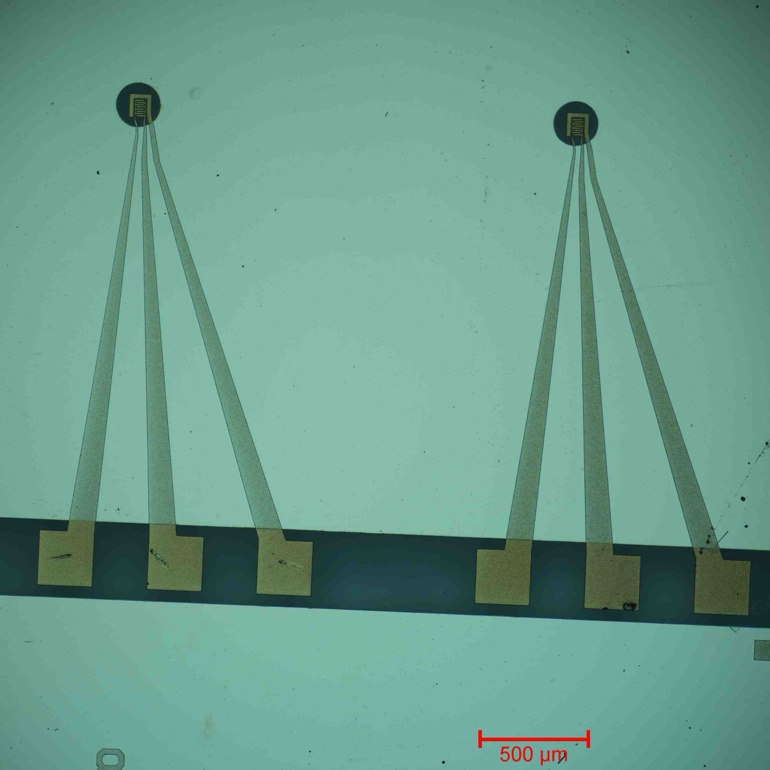
Photolithographic Masking of Sensing Region and Contact Pads Prior to Micropillar Fabrication*

Figure S. 1. Photolithographic masking of the sensing region and contact pads to prevent micropillar growth. Openings were patterned to expose these areas for selective Cr/W and Al_2_O_3_ removal, ensuring pillars form only in designated superhydrophobic zones.

1. *
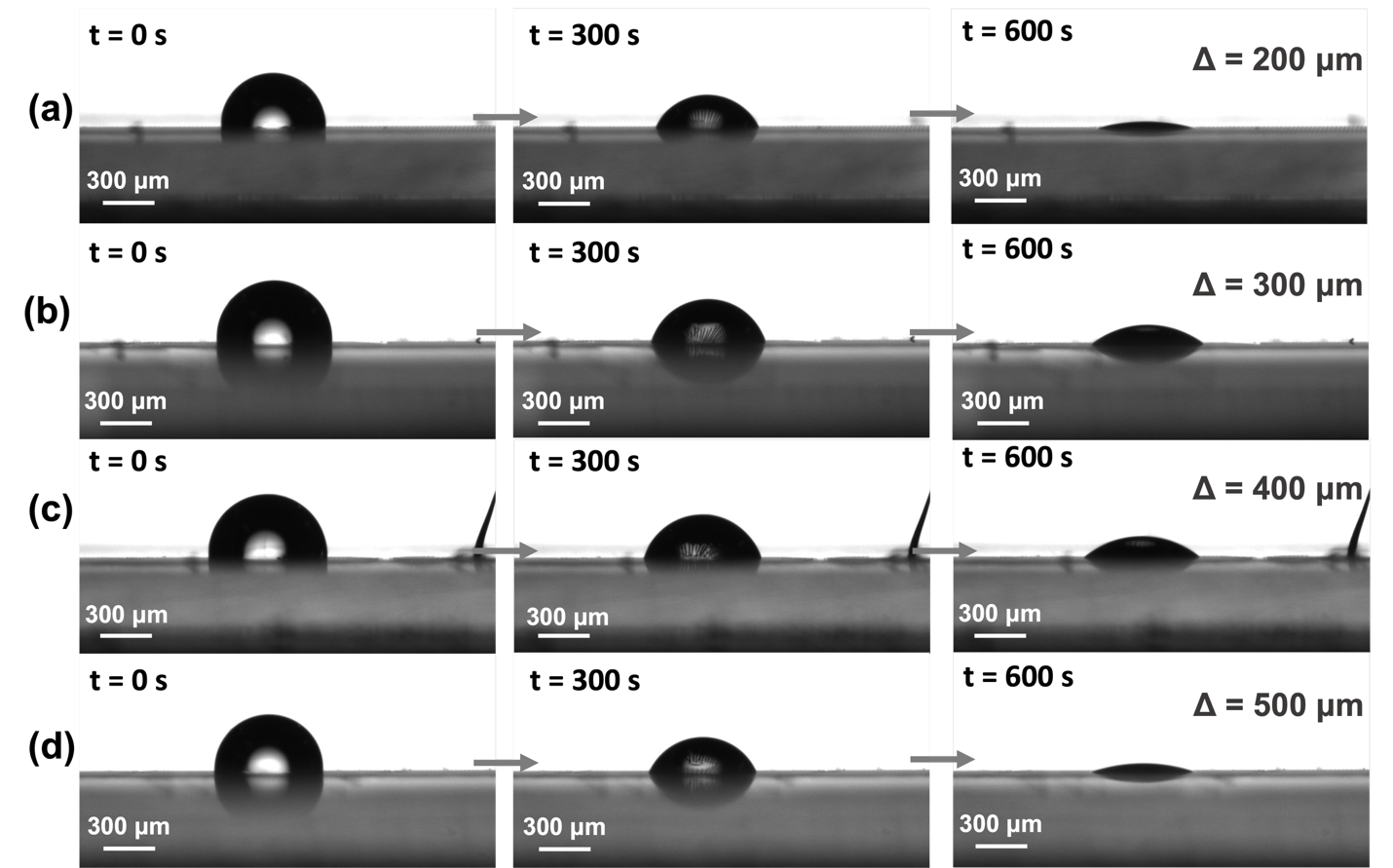
Wettability contrast between superhydrophobic micropillar regions and hydrophilic sensing zone in SW-E2RC devices.*

Figure S. 2. Apparent contact angle measurement on the integrated SW-E2RC surface, highlighting the wettability unstructured central sensing zone (~$100^{\circ}$). While the micropillars provide extreme water repellency to confine the droplet, the smooth hydrophilic sensing area enables direct electrochemical access for redox cycling, resulting in a reduced overall apparent contact angle for the combined surface.

1. *
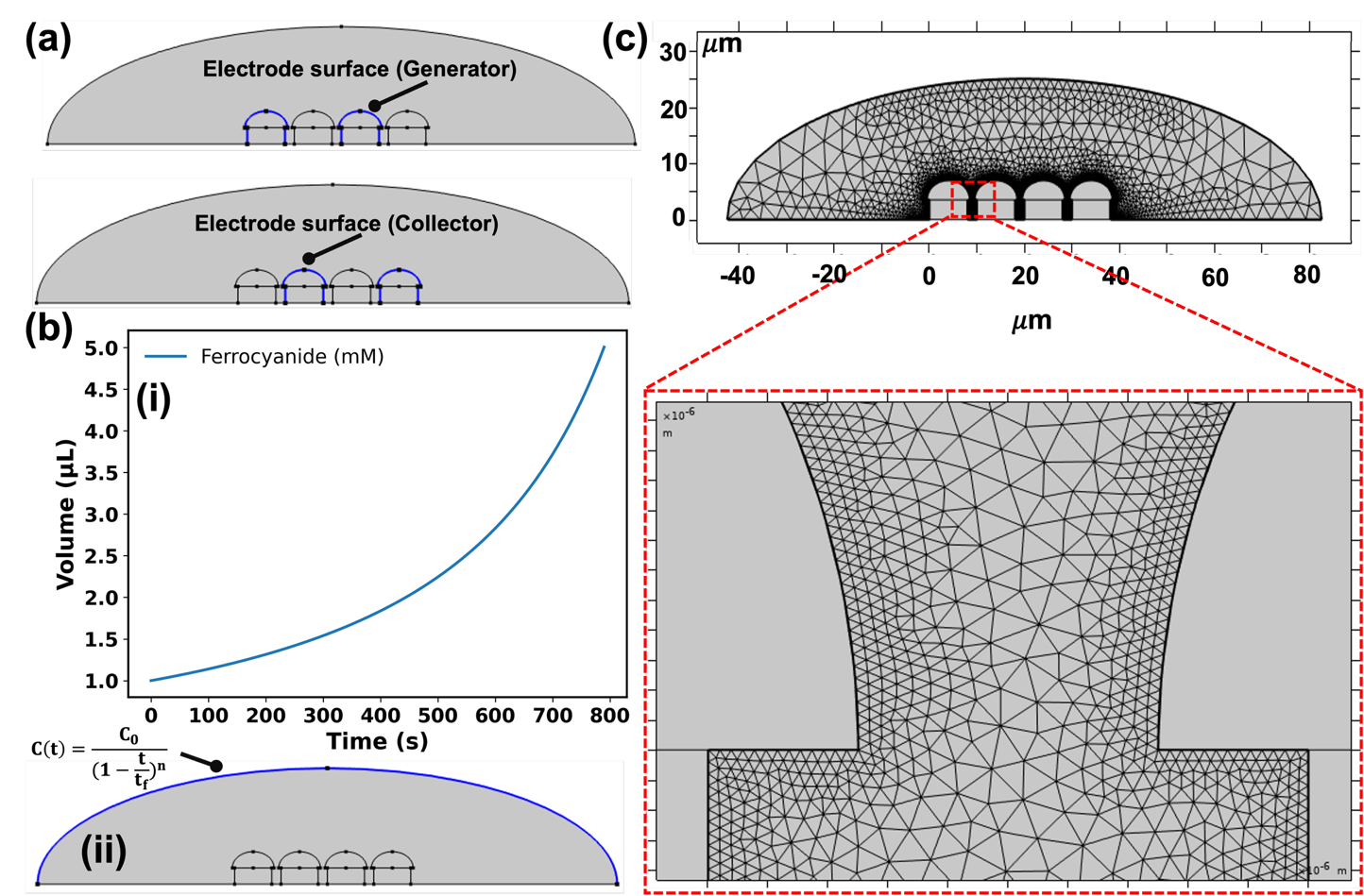
Mesh Refinement Strategy for Ensuring Grid Independence in Finite Element Analysis*

Figure S. 3. **(a)** Geometry of the 2D axisymmetric COMSOL simulation domain used for modeling SW-E2RC, consisting of two coplanar electrodes: a generator (swept from –1.0 to +1.0 V at 50 mV s⁻¹ for 10 cycles) and a collector (held at –1.0 V), submerged in 10 mM KCl electrolyte with 1 mM [Fe(CN)₆]⁴⁻. (b) (i) Time-dependent increase in redox molecule concentration due to evaporation-induced enrichment modeled using a power-law decay function for droplet volume; (ii) this enriched concentration field was applied at the droplet-electrolyte interface to drive current amplification. (c) Finite element mesh used in the simulation, showing local refinement around electrode edges and inter-electrode gaps to resolve high-gradient zones in current density and potential.

1. *
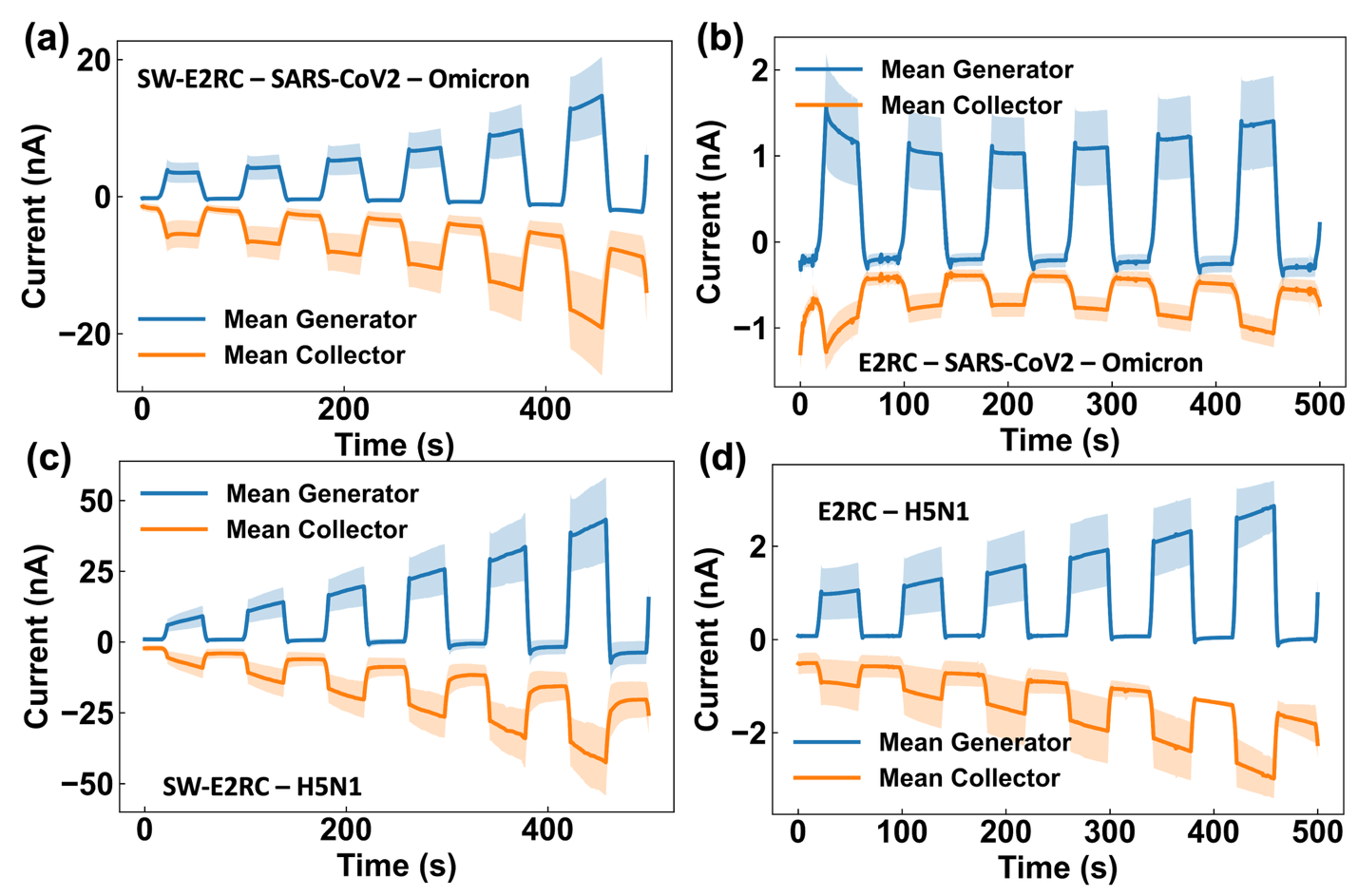
* ***Peak current evolution in SW-E2RC versus E2RC***

Figure S. 4. (**a, b**) SARS-CoV-2 Omicron ($7.28\times{10}^{7} copies/mL$), (**c, d**) H5N1 ($7.28\times{10}^{7} copies/mL$). Peak generator and collector currents extracted from the time profiles in Figure 4 highlight the consistent amplification achieved by SW-E2RC compared to conventional E2RC. The integration of selective wettability ensures amplified current responses and reduced variability across replicates, confirming the role of droplet confinement in improving redox cycling performance. Data shown as mean ± SD ($n=3$).

1. *
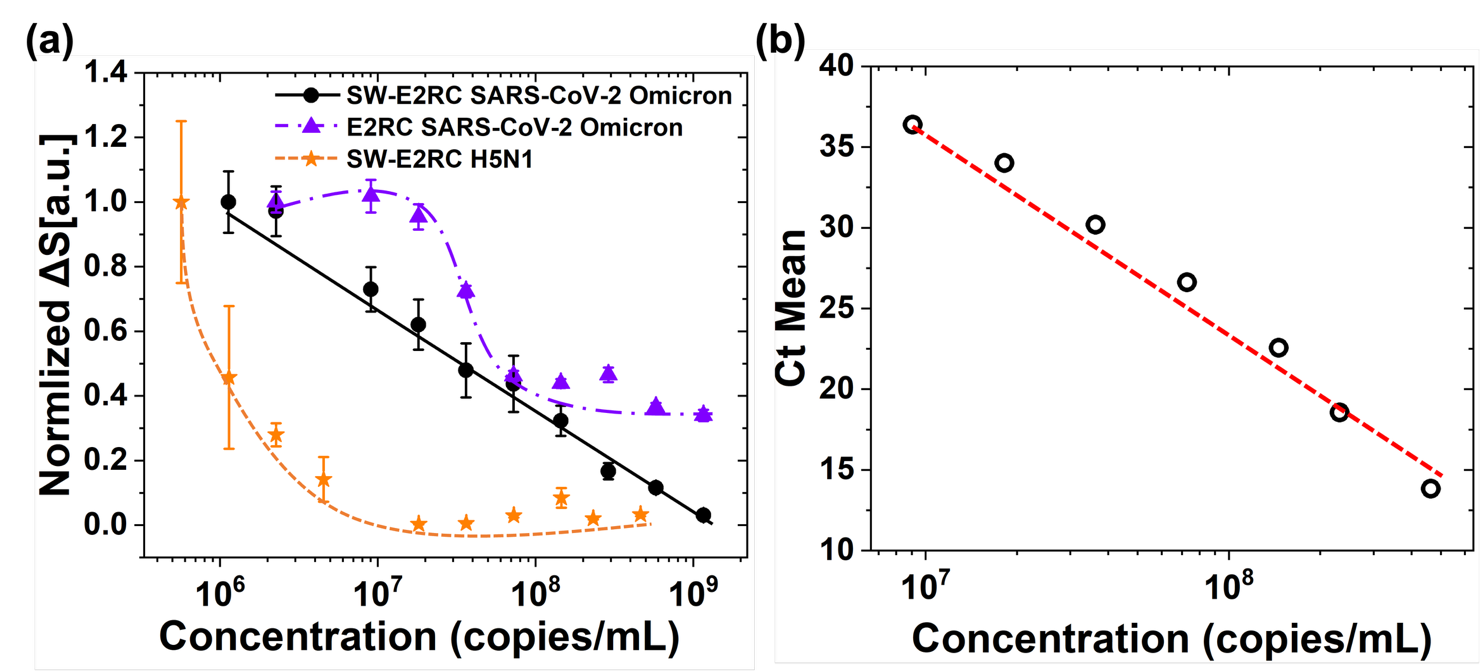
 ΔS comparison for H5N1 detection using SW-E2RC and E2RC, showing performance gains from selective wettability integration.*

Figure S. 5. **(a)** Normalized $\Delta S$ versus viral concentration for SW-E2RC and E2RC platforms detecting SARS-CoV-2 Omicron and SW-E2RC detecting H5N1 virus. Data shown as mean $\pm$SE (n=9). **(b)** RT-PCR cycle threshold (Ct) values for the H5N1 samples.
